# Spin- and Valley-Dependent Electronic Structure in Silicene Under Periodic Potentials

**DOI:** 10.1186/s11671-018-2495-4

**Published:** 2018-03-23

**Authors:** Wei-Tao Lu, Yun-Fang Li, Hong-Yu Tian

**Affiliations:** 1 School of Physics and Electronic Engineering, Linyi University, Linyi, 276005 China; 2 School of Mechanical & Vehicle Engineering, Linyi University, Linyi, 276005 China

**Keywords:** Silicene, Energy band, Valley polarization, Spin polarization

## Abstract

We study the spin- and valley-dependent energy band and transport property of silicene under a periodic potential, where both spin and valley degeneracies are lifted. It is found that the Dirac point, miniband, band gap, anisotropic velocity, and conductance strongly depend on the spin and valley indices. The extra Dirac points appear as the voltage potential increases, the critical values of which are different for electron with different spins and valleys. Interestingly, the velocity is greatly suppressed due to the electric field and exchange field, other than the gapless graphene. It is possible to achieve an excellent collimation effect for a specific spin near a specific valley. The spin- and valley-dependent band structure can be used to adjust the transport, and perfect transmissions are observed at Dirac points. Therefore, a remarkable spin and valley polarization is achieved which can be switched effectively by the structural parameters. Importantly, the spin and valley polarizations are greatly enhanced by the disorder of the periodic potential.

## Background

Two-dimensional (2D) Dirac materials with hexagonal lattice structures are being explored extensively since the discovery of graphene, such as silicene [1, 2], transition metal dichalcogenides [3, 4], and phosphorene [5]. Although graphene has many particular properties, its application is limited by the zero band gap and the weak spin-orbit interaction (SOI). Recently, a silicon analog of graphene, silicene, has been fabricated via epitaxial growth [6–10], and its stability has been predicted by theoretical studies [11, 12]. Graphene and silicene have similar band structures around *K* and *K*^′^ valleys, and the low energy spectra of both are described by the relativistic Dirac equation [13]. Contrary to graphene, silicene has a strong intrinsic SOI and a buckled structure. The strong SOI could open a gap at Dirac points [13, 14] and lead to a coupling between the spin and valley degrees of freedom. The buckled structure allows us to control the band gap by an external electric field perpendicular to the silicene sheet [14–16]. Furthermore, silicene has the advantage that it is more compatible with existing silicon-based electronic technology. These characteristics make silicene an excellent material for the next-generation nanoelectronics. In particular, a silicene field-effect transistor at room temperature has been successfully fabricated by a growth-transfer-fabrication process in experiment [17].

The discovery of 2D Dirac materials provides new opportunities to explore quantum control of valley. The two inequivalent valleys *K* and *K*^′^ in the first Brillouin zone could be regarded as an additional degree of freedom besides charge and spin for quantum information and quantum computation [18–20]. For example, the valley degree of freedom can be incorporated to expand an electron spin qubit to a spin-valley qubit [18]. Therefore, valleytronics which aims to generate, detect, and manipulate the valley pseudospin has attracted considerable interest. In graphene, various schemes to achieve a valley polarization have been proposed by utilizing unique edge modes [21, 22], trigonal warping effect [23], topological line defects [24, 25], strain [26, 27], and electrostatic gates [28]. Compared to graphene, silicene has significant advantage in the study of valley pseudospin. It is found that silicene exhibits a rich variety of topological phases and Chern numbers under the modulation of different external fields [13, 16, 29, 30]. In the presence of electric field *E*_*z*_ and exchange field *h*, Ezawa explored the phase diagram in the *E*_*z*_−*h* plane which is characterized by the spin and valley indices [16]. Further considering the Rashba SOI, a valley-polarized quantum anomalous Hall state is predicted in silicene owing to the topological phase transition [31]. Based on the state transition, a silicene-based spin filter with nearly 100% spin polarization is proposed which is robust against weak disorder [32]. Yokoyama studied the ballistic transport through a ferromagnetic (FM) silicene junction and demonstrated a controllable spin and valley polarized current [33]. In transition metal dichalcogenides with a broken inversion symmetry, the spin splitting of the valence bands arising from intrinsic SOI is opposite at the two valleys due to a time-reversal symmetry [3, 34, 35]. The broken inversion symmetry could result in a valley-dependent optical selection rule, which can be used to selectively excite carriers in the *K* or *K*^′^ valley via right or left circularly polarized light, respectively [3, 34]. In experiment, the signal of valley polarization has been probed by optical [36, 37] and transport [38, 39] measurements. A giant nonlocal valley Hall effect was observed in bilayer graphene subjected to a symmetry-breaking gate electric field, and the nonlocal signal persists up to room temperature [38]. A recent review of valleytronics in 2D Dirac materials is provided in Ref. [40].

Superlattice is an effective method of engineering the electronic structure in semiconductors and 2D materials [41]. Superlattice patterns with nanoscale can naturally arise in experiment when graphene or silicene is placed on top of metallic substrates [42, 43]. A superlattice in graphene could lead to renormalization of anisotropic Fermi velocity [44] and generation of new Dirac points in the spectrum [45–47] owing to the chiral nature, which have been experimentally observed [43, 48, 49]. In silicene superlattices with electric field *E*_*z*_ and exchange field *h*, both spin and valley degeneracies are lifted. It is confirmed that miniband structure and minigaps caused by the superlattices depend on the spin and valley indices [50]. Furthermore, the spin and valley polarizations could be enhanced by the silicene superlattices [51]. Just like graphene, many novel electronic structures are expected in silicene superlattices. However, works on silicene superlattices are very few [50, 51]. In this paper, we discuss in detail a complementary aspect, namely, the spin- and valley-dependent band structure and transport property of silicene. We found that the spin and valley indices have different impacts on the extra Dirac points and anisotropic velocity which can be tuned by the structural parameters. The velocity is greatly suppressed due to the electric field and exchange field. A remarkable spin and valley polarization is achieved, which can be greatly enhanced by the disorder.

The paper is organized as follows. In the “Methods” section, we present the theoretical formalism and the dispersion relation. The numerical results on band structure, spin and valley polarized transmissions are shown in the “Results and discussions” section. Finally, we conclude with a summary in “Conclusions” section.

## Methods

**Fig. 1:**
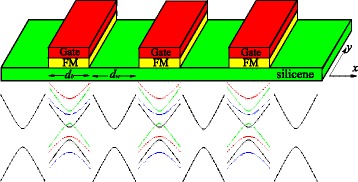
Top: schematic of the silicene superlattices. The FM insulators, such as EuO and EuS, on the top of silicene induce the exchange fields in silicene, as proposed for graphene [53]. The metallic gates on the top of FM insulators control the Fermi level locally. Bottom: schematic of the energy spectrum in silicene with and without external fields

In the single-particle approximation, the electronic structure of silicene in the vicinity of Dirac points obeys an effective Dirac Hamiltonian. The system under consideration is a one-dimensional silicene superlattice formed by a series of local potential barriers *U*, exchange fields *h*, and perpendicular electric field *E*_*z*_. *U*, *h*, and *E*_*z*_ are present only in the barrier regions with barrier width *d*_*b*_, whereas *U*=*h*=*E*_*z*_=0 in the well regions with well width *d*_*w*_, as shown in Fig. 1. The superlattice with a Kronig-Penney type varies only along *x* direction, and the length of one unit is *d*=*d*_*b*_+*d*_*w*_. Similar model has been discussed in Refs. [51, 52], which mainly focus on thermoelectric and electronic transport rather than the band structure and disorder effect studied in this work. Experimentally, *U* can be produced by the metallic gates and *h* can be produced by the magnetic proximity effect with FM insulators EuO [33], which are deposited periodically on top of the silicene layer (see Fig. 1). The electric field *E*_*z*_ applied perpendicular to silicene can induce a staggered sublattice potential *Δ*_*z*_=*ℓE*_*z*_, with 2*ℓ*≈0.46Å the vertical separation of *A* and *B* sites of the two sublattices due to the buckled structure [16]. Hence, the electronic states can be described by the Hamiltonian,

1 $$\begin{array}{@{}rcl@{}} H = \hbar v_{F} (k_{x} \tau_{x} - \eta k_{y} \tau_{y}) + \Delta_{\eta \sigma} \tau_{z} + U_{\sigma}. \end{array} $$

*Δ*_*ησ*_=*Δ*_*z*_−*ησλ*_*SO*_ describes the band gap for different spin and valley indices, which can be controlled by the staggered potential *Δ*_*z*_ and the SOI *λ*_*SO*_. *U*_*σ*_=*U*−*σh* is the effective potential for different spin indices. *η*=±1 denotes the *K* and *K*^′^ valleys. *σ*=±1 denotes spin-up and spin-down states. *v*_*F*_ is the Fermi velocity. In silicene, the intrinsic and extrinsic Rashba effects are very small and can be neglected [15].

Due to the translational invariance along the *y* direction, the transverse wave vector *k*_*y*_ is conserved. The wave function for valley *η* and spin *σ* in each region has the form *Ψ*(*x*,*y*)=*ψ*(*x*)*e*^*iky*^_*y*_ with

2 $$\begin{array}{@{}rcl@{}} \psi(x) = A \left(\begin{array}{cc} 1 \\ \frac{\hbar v_{F} k_{-}}{\epsilon_{\eta \sigma}} \end{array}\right) e^{i q_{\eta \sigma} x} + B \left(\begin{array}{cc} 1 \\ \frac{- \hbar v_{F} k_{+}}{\epsilon_{\eta \sigma}} \end{array}\right) e^{-i q_{\eta \sigma} x}. \end{array} $$

In the barrier regions, *ε*_*ησ*_=*ε*_*b*_=(*E*−*U*_*σ*_)+*Δ*_*ησ*_ and the *x* component of the wave vector $ q_{\eta \sigma } = q_{b} = \sqrt {(E - U_{\sigma })^{2} - \Delta ^{2}_{\eta \sigma } - (\hbar v_{F} k_{y})^{2}} / \hbar v_{F} $. In the well regions, *ε*_*ησ*_=*ε*_*w*_=*E*−*ησλ*_*SO*_ and $ q_{\eta \sigma } = q_{w} = \sqrt {E^{2} - \lambda _{SO}^{2} - (\hbar v_{F} k_{y})^{2}} / \hbar v_{F} $. *k*_±_=*q*_*ησ*_±*iηk*_*y*_. The transmission probability *T*_*ησ*_ can be calculated using the transfer matrix technique. The normalized conductance for a particular spin in a particular valley at zero temperature is given by

3 $$\begin{array}{@{}rcl@{}} G_{\eta \sigma} (E) = \frac{1}{2} \int_{-\pi/2}^{\pi/2} T_{\eta \sigma} (E,E\sin \theta) \cos \theta d \theta, \end{array} $$

where *θ* is the incident angle with respect to the *x* direction. The spin- and valley-resolved conductances are defined as $G_{\uparrow (\downarrow)} = \left (G_{K \uparrow (\downarrow)} + G_{K^{\prime } \uparrow (\downarrow)} \right) / 2 $ and $G_{K (K^{\prime })} = \left (G_{K (K^{\prime }) \uparrow } + G_{K (K^{\prime }) \downarrow } \right) / 2$, respectively. Then, we introduce the spin polarization *P*_*s*_ and valley polarization *P*_*v*_:

4 $$\begin{array}{@{}rcl@{}} P_{s} = (G_{\uparrow} - G_{\downarrow}) / (G_{\uparrow} + G_{\downarrow}), \end{array} $$

5 $$\begin{array}{@{}rcl@{}} P_{v} = (G_{K} - G_{K^{\prime}}) / (G_{K} + G_{K^{\prime}}). \end{array} $$

Based on the Bloch’s theorem and the continuity condition of wave functions, the dispersion relation *E*(*k*_*x*_) for spin-up and spin-down electrons near the *K* and *K*^′^ valleys can be calculated,

6 $$ \begin{aligned} \cos(k_{x} d) &= \cos(q_{w} d_{w}) \cos(q_{b} d_{b}) \\&\quad- \frac{(\epsilon_{b} q_{w})^{2} + (\epsilon_{w} q_{b})^{2} + (\epsilon_{b} \!- \!\epsilon_{w})^{2} k^{2}_{y}}{2 \epsilon_{w} \epsilon_{b} q_{w} q_{b}} \sin(q_{w} d_{w}) \sin(q_{b} d_{b}), \end{aligned}  $$

and *k*_*x*_ is the Bloch wave number. In order to simplify the calculation, the dimensionless units are introduced: $E \rightarrow E d / \hbar v_{F}$, $U \rightarrow U d / \hbar v_{F}$, $\lambda _{SO} \rightarrow \lambda _{SO} d / \hbar v_{F}$, $\Delta _{z} \rightarrow \Delta _{z} d / \hbar v_{F}$, $h \rightarrow h d / \hbar v_{F}$, *k*_*y*_→*k*_*y*_*d*, *k*_*x*_→*k*_*x*_*d*, *d*_*w*_→*d*_*w*_/*d*, and *d*_*b*_→*d*_*b*_/*d*. Note that at *Δ*_*z*_=*λ*_*SO*_=*h*=0, Eq. (6) is reduced to the one found for gapless graphene in a periodic potential, where both spin and valley are degenerated [44–47]. From Eq. (6), we can see that exchange field *h* alone could induce the split of spin, while the valley keeps degeneracy. However, the valley degeneracy can be lifted by the electric field *E*_*z*_ with the help of the SOI *λ*_*SO*_. Thus, a combination of the exchange field and the electric field could lift the spin and valley degeneracies [16, 31–33], as shown in Fig. 1. In the proposed system, electrons with different spins near different valleys would present various band structures and transport features.

## Results and discussions

In this section, we would use the above equations to calculate the band structures and transport properties for different spin and valley indices in silicene superlattices. The widths of barriers and wells are assumed to be the same in what follows. The results for the case with unequal well and barrier widths (*d*_*b*_≠*d*_*w*_) are similar to those in gapless graphene [47]. Some parameters are set as *d*_*b*_=*d*_*w*_=50 nm and *λ*_*SO*_=3.9 meV in silicene, unless otherwise stated. We shall concentrate on the first two minibands (the lowest valence and conduction minibands) near the Fermi level.

### Spin- and Valley-Dependent Band Structure

**Fig. 2:**
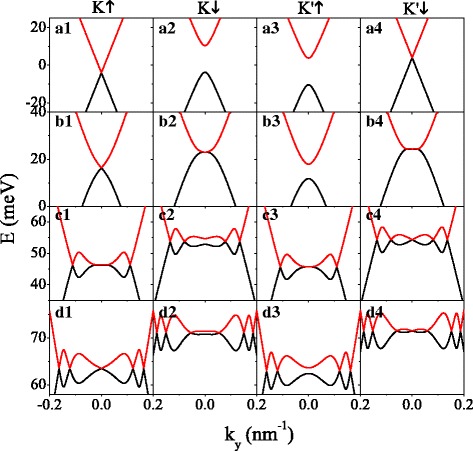
Energy spectrum versus *k*_*y*_ for several different values of potential *U*. (a1–a4) *U*=0; (b1–b4) *U*=40.66 meV; (c1–c4) *U*=100.63 meV; (d1–d4) *U*=135.0 meV. The values of parameters are *h*=8.0 meV, *Δ*_*z*_=7.8 meV, and *k*_*x*_=0

First, the effect of potential *U* on minibands is depicted in Fig. 2. In order to discuss the gapped case and gapless case of energy bands simultaneously, we set *Δ*_*z*_=7.8 meV=2*λ*_*SO*_. In the absence of potential (*U*=0), the spin-up electron near *K* valley (*K↑* electron) and spin-down electron near *K*^′^ valley (*K*^′^*↓* electron) are gapless (see Fig. 2 (a1, a4)), while the spin-down electron near *K* valley (*K↓* electron) and spin-up electron near *K*^′^ valley (*K*^′^*↑* electron) have a large gap (see Fig. 2 (a2, a3)). The minibands of spin-up (or spin-down) electron shift to the negative (or positive) energy range from *E*=0 by *h*, due to the effective potential *U*_*σ*_=*U*−*σh*. The band structures of *K↑* (or *K↓*) electron and *K*^′^*↓* (or *K*^′^*↑*) electron present mirror symmetry with respect to *E*=0, consistent with Eq. (6). However, this mirror symmetry is destroyed in the presence of *U*. Observably, as *U* increases, extra Dirac points appear, the number of which increases in the meantime. The extra Dirac points can be demonstrated by the chirality of the wave functions in their vicinity [46]. The features of Dirac points in silicene system rely heavily on the spin and valley degrees of freedom, as shown in Fig. 2. For example, at *U*=135 meV in Fig. 2 (d1–d4) for *K↑*, *K↓*, *K*^′^*↑* and *K*^′^*↓* electrons, the numbers of Dirac points are 5, 6, 4, and 7, respectively. For specific values of *U*, such as *U*=40.66 meV for *K↓* electron (see Fig. 2 (b2)) and *U*=100.63 meV for *K*^′^*↑* electron (see Fig. 2 (c3)), a new Dirac point can be generated at *k*_*y*_=0, and it will split into a pair which move in opposite directions away from the *k*_*y*_=0 point but always keeping *k*_*x*_=0, as *U* further increases. In consequence, the band gaps for *K↓* and *K*^′^*↑* electrons are closed (see Fig. 2 (b2, c3)), and the gapped system becomes gapless. In order to find the critical value of *U*, we set *d*_*b*_=*d*_*w*_ and *k*_*x*_=0. Analogous to the rule in gapless graphene [47], taking into account the implicit function theorem, one can conclude that the longitudinal wavevectors at the new Dirac points satisfy *q*_*b*_=*q*_*w*_ when

7 $$\begin{array}{@{}rcl@{}} E_{0} = \frac{(U - \sigma h)^{2} - \Delta_{z}^{2} + 2 \eta \sigma \Delta_{z} \lambda_{SO}}{2(U - \sigma h)}. \end{array} $$

For *K↑* and *K*^′^*↓* electrons with *ησ*=1, when *Δ*_*z*_=2*λ*_*SO*_, Eq. (7) can reduce to

8 $$\begin{array}{@{}rcl@{}} E_{0} = \frac{U - \sigma h}{2}. \end{array} $$

Correspondingly, Eq. (6) turns into

9 $$ {}\cos^{2}(q_{w} d_{w}) - \frac{\left(\epsilon_{b}^{2} + \epsilon_{w}^{2}\right) q_{w}^{2} + (\epsilon_{b} - \epsilon_{w})^{2} k^{2}_{y}}{2 \epsilon_{w} \epsilon_{b} q_{w}^{2}} \sin^{2}(q_{w} d_{w}) = 1,  $$

which is satisfied when $\left (\epsilon _{b}^{2} + \epsilon _{w}^{2}\right) q_{w}^{2} + (\epsilon _{b} - \epsilon _{w})^{2} k^{2}_{y} = -2 \epsilon _{w} \epsilon _{b} q_{w}^{2}$ or *q*_*w*_*d*=2*nπ* (*n* is a positive integer). Based on Eq. (8), we have *ε*_*b*_=−*ε*_*w*_, and so the former equality is fulfilled only if *k*_*y*0_=0 for *K↑* and *K*^′^*↓* electrons at *Δ*_*z*_=2*λ*_*SO*_, corresponding to the original Dirac point. The solutions of *q*_*w*_*d*=2*nπ* have the form

10 $$\begin{array}{@{}rcl@{}} k_{y0} = \pm \frac{1}{d} \sqrt{\frac{\left(E_{0}^{2}-\lambda_{SO}^{2}\right)d^{2}}{(\hbar v_{F})^{2}} - (2n\pi)^{2}}. \end{array} $$

When $\sqrt {E_{0}^{2}-\lambda _{SO}^{2}}d / 2\pi \hbar v_{F} \geq n$, *k*_*y*0_ is real, and the new Dirac points will arise which are exactly located at (*E*_0_,*k*_*y*0_). At low values of *U*, *k*_*y*0_ is imaginary, and there is no solution for *n*, which means no extra Dirac points. The Dirac points appear only after a critical value of *U*, such as *U*=40.66 meV for *K↓* electrons in Fig. 2 (b2), corresponding to *n*=1. According to Eq. (10), The number of Dirac points *N*_*D*_ can be obtained. When *Δ*_*z*_=2*λ*_*SO*_,

11 $$\begin{array}{@{}rcl@{}} N_{D} = 2 \left[ \frac{\sqrt{E_{0}^{2}-\lambda_{SO}^{2}}d}{2\pi\hbar v_{F}} \right] + 1 \end{array} $$

for *K↑* and *K*^′^*↓* electrons, while

12 $$\begin{array}{@{}rcl@{}} N_{D} = 2 \left[ \frac{\sqrt{E_{0}^{2}-\lambda_{SO}^{2}}d}{2\pi\hbar v_{F}} \right] \end{array} $$

for *K↓* and *K*^′^*↑* electrons, where [...] denotes an integer part. Note that at the critical value of *U*, such as *U*=40.66 meV and 100.63 meV, the number of Dirac points is *N*_*D*_=2*n*−1 for *K↓* and *K*^′^*↑* electrons (see Fig. 2 (b2, c3)).

**Fig. 3:**
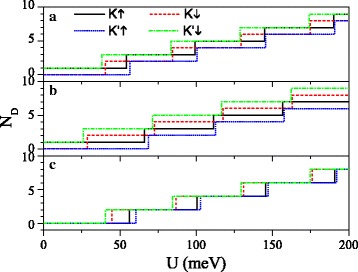
Number of Dirac points *N*_*D*_ versus potential *U*. (**a**) *h*=8.0 meV and *Δ*_*z*_ = 7.8 meV; (**b**) *h* = 20.0 meV and *Δ*_*z*_ = 7.8 meV; (**c**) *h* = 8.0 meV and *Δ*_*z*_=15.0 meV

Equations (7) and (10) manifest that the positions and the numbers of Dirac points could be adjusted by the electric field *E*_*z*_ and exchange field *h*. Figure 3 exhibits the number of Dirac points *N*_*D*_ as a function of *U* for different values of *E*_*z*_ and *h*. When *Δ*_*z*_=7.8 meV in Fig. 3a, with increasing *U*, *N*_*D*_ for *K↑* and *K*^′^*↓* electrons increases in the form of odd number, consistent with Eq. (11). *N*_*D*_ for *K↓* and *K*^′^*↑* electrons increases in the form of even number, consistent with Eq. (12), except for *N*_*D*_ at the critical value. Comparison between Fig. 3a and b indicates that as *h* increases, the critical value for spin-down (or spin-up) electron decreases (or increases) gradually. When *Δ*_*z*_=15 meV≠2*λ*_*SO*_ in Fig. 3c, *N*_*D*_ for all electrons increases in the form of even number, except for *N*_*D*_ at the critical value. Distinctly, the critical values of *U* are different for electron with different spins and valleys. The Dirac points could be controlled by a joint modulation of the parameters *U*, *E*_*z*_, and *h*.

**Fig. 4:**
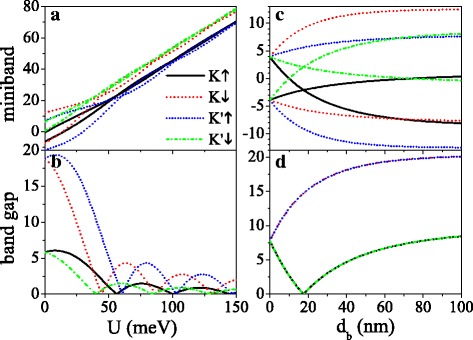
(**a**) Minibands near Fermi level and (**b**) their band gaps at original Dirac point versus potential *U*, at *d*_*b*_=*d*_*w*_=50 nm. (**c**) Minibands near Fermi level and (**d**) their band gaps at original Dirac point versus *d*_*b*_, at *U*=0 and *d*_*w*_=50 nm. The values of other parameters are *h*=8.0 meV, *Δ*_*z*_=15.0 meV, and *k*_*x*_=*k*_*y*_=0

The potential *U* and barrier width *d*_*b*_ could be used to regulate the band gap, as illustrated in Fig. 4. The gaps for *K↑* and *K*^′^*↓* electrons are small, while the gaps for *K↓* and *K*^′^*↑* electrons are large due to *Δ*_*ησ*_=*Δ*_*z*_−*ησλ*_*SO*_. As *U* increases, all the minibands gradually move toward high energy region (see Fig. 4a), and all the band gaps display damped oscillation with *U* (see Fig. 4b). When *U*=*σh*, the effective potential is zero, and the gap reaches maximum value. The gap is closed at the critical value of *U*, on account of the emergence of new Dirac points. Figure 4c, d depicts the dependence of minibands and band gaps on barrier width *d*_*b*_ at *U*=0. In the absence of external field (*d*_*b*_=0), the minibands keep degenerate, and the gap at Fermi level is 2*λ*_*SO*_. With the appearance of *d*_*b*_, the miniband is split, where valley and spin become nondegenerate. The minibands of *K↑* (or *K↓*) and *K*^′^*↓* (or *K*^′^*↑*) electrons keep mirror symmetry about *E*=0 (see Fig. 4c). As *d*_*b*_ increases, the gaps of *K↓* and *K*^′^*↑* electrons are broaden gradually. The gaps of *K↑* and *K*^′^*↓* electrons decrease to zero when *d*_*b*_ satisfies *d*_*b*_/*d*_*w*_=*λ*_*SO*_/*Δ*_*z*_, and thereafter increase with *d*_*b*_ (see Fig. 4d). The gap widths approach to saturation with the further increase of *d*_*b*_. Furthermore, the width of miniband is narrowed as *d*_*b*_ increases (not shown in the figure), due to the less coupling of eigenstates. The effect of electric field on band gap is analogous to that in previous study [50].

**Fig. 5:**
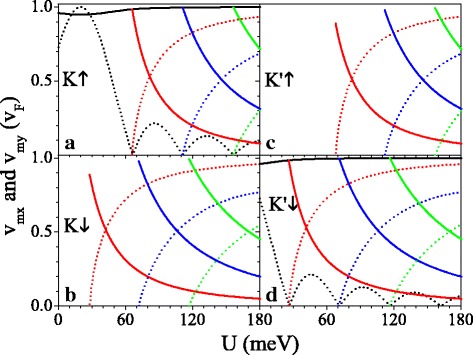
(**a**–**d**) Velocity versus potential *U*, and the parameters are set as *h*=20.0 meV and *Δ*_*z*_=7.8 meV. The black, red, blue, and green solid curves are the velocities *v*_0*x*_, *v*_1*x*_, *v*_2*x*_, and *v*_3*x*_, respectively. The black, red, blue, and green dashed curves are the velocities *v*_0*y*_, *v*_1*y*_, *v*_2*y*_, and *v*_3*y*_, respectively

The group velocity depends strongly on the spin and valley indices, as shown in Fig. 5. The components (*v*_*x*_,*v*_*y*_) of velocity can be defined as

13 $$\begin{array}{@{}rcl@{}} v_{x} / v_{F} = \partial E / \partial k_{x}, \quad v_{y} / v_{F} = \partial E / \partial k_{y}. \end{array} $$

**Fig. 6:**
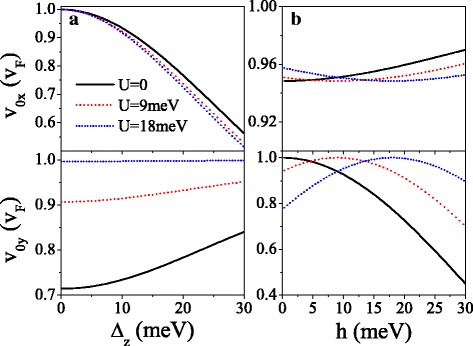
Velocities *v*_0*x*_ and *v*_0*y*_ versus (**a**) *Δ*_*z*_ and (**b**) *h*, for *K↑* electron. (**a**) *h*=20.0 meV and *λ*_*SO*_=*Δ*_*z*_/2. (**b**) *Δ*_*z*_=7.8 meV

Figure 5 presents the velocity components *v*_*mx*_ and *v*_*my*_ in units of *v*_*F*_ at original Dirac point (*m*=0) and new Dirac points (*m*=1,2,3). One can see that as *U* increases, *v*_0*y*_ oscillates in a decayed way and *v*_0*x*_≈*v*_*F*_ is almost unaffected (see Fig. 5a, d). At the critical value of *U* where the new Dirac points emerge, *v*_*mx*_≈*v*_*F*_ but *v*_0*y*_=*v*_*my*_=0, indicating a collimation behavior along the *k*_*x*_ direction for specific spins and valleys. When *U* exceeds the critical value and further increases, *v*_*my*_ increase to *v*_*F*_ but *v*_*mx*_ decrease to zero gradually. The effect of the periodic potential is highly anisotropic, as a result of the chiral nature. The features of anisotropic velocity are various for different spins and valleys owing to the gap *Δ*_*ησ*_ and the potential *U*_*σ*_, which can be commanded by employing *U*. Taking *U*=20 meV for example, *v*_0*y*_=*v*_*F*_ for *K↑* electron is much greater than *v*_0*y*_=0.16*v*_*F*_ for *K*^′^*↓* electron, and no *v*_0*y*_ for *K↓* and *K*^′^*↑* electrons due to the band gap. *v*_*mx*_ (or *v*_*my*_) for spin-up electron is always larger (or less) than the one for spin-down electron in the same valley. Notably, Fig. 5 also implies that for small value of *U*, *v*_0*x*_, *v*_0*y*_, and *v*_*mx*_ are less than *v*_*F*_ due to *Δ*_*z*_ and *h*, other than the gapless system [44]. For instance, *v*_1*x*_=0.98*v*_*F*_, 0.89*v*_*F*_, 0.89*v*_*F*_, and 0.98*v*_*F*_ for *K↑*, *K↓*, *K*^′^*↑* and *K*^′^*↓* electrons, respectively, when the Dirac point appears. In order to illuminate the influence of *Δ*_*z*_ and *h* on the group velocity, Fig. 6 shows the velocities (*v*_0*x*_,*v*_0*y*_) as a function of (a) *Δ*_*z*_ and (b) *h* for *K↑* electron. From Fig. 6a we can clearly see that *v*_0*x*_ is monotonically decreasing with *Δ*_*z*_ while *v*_0*y*_ is insensitive to the change of *Δ*_*z*_. On the contrary, *v*_0*x*_ is desensitized to *h*, while *v*_0*y*_ increases to maximum value *v*_0*y*_=*v*_*F*_ at *h*=*σU* and then decreases with *h*. The results indicate that the group velocity can be suppressed by *Δ*_*z*_ and *h* in silicene.

### Spin- and Valley-Polarized Transport

**Fig. 7:**
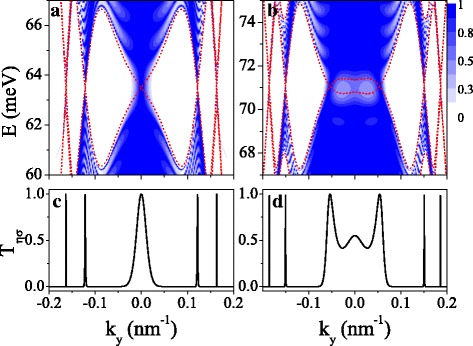
Contour plot of the transmission *T*_*ησ*_(*E*,*k*_*y*_) for (**a**), (**c**) *K↑* electron and (**b**), (**d**) *K↓* electron. The values of parameters are the same as these in Fig. 2 (d1–d4), and *n*=10

The spin- and valley-dependent band structure is reflected in transport property and provides a guide in controlling the transport. In this section, we discuss the properties of spin- and valley-polarized transport through a finite silicene superlattice. Figure 7 shows the transmission probability *T*_*ησ*_ for (a, c) *K↑* and (b, d) *K↓* electrons, and the period number *n*=10. The red dashed curves are the minibands, which are also the borders for different electronic states deciding the transmission. We can see that the transmission is restricted in the miniband region and no transmission in the band gap region (see Fig. 7a, b). The distribution of transmission is symmetric around *k*_*y*_=0 due to the symmetric minibands. The resonant characteristic of transmission arises from the resonant states. It should be noted that the transmission still exists in the gap region near *k*_*y*_=0 due to the tunnel effect of eigenstates. *T*_*ησ*_ at Fermi level for *K↑* and *K↓* electrons are shown in Fig. 7c, d), respectively. One can clearly see that many thin resonant peaks with *T*_*ησ*_=1 occur precisely at the positions of the Dirac points, suggesting an application of the system as a spin and valley filter.

**Fig. 8:**
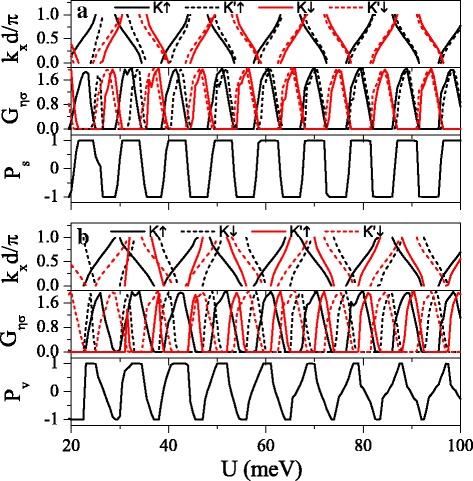
Minibands, conductances *G*_*ησ*_, spin polarization *P*_*s*_, and valley polarization *P*_*v*_ versus potential *U*. (**a**) *Δ*_*z*_=4.0 meV. (**b**) *Δ*_*z*_=12.0 meV. Other parameters are set as *h*=7.0 meV, *E*=6.0 meV, *d*_*b*_=*d*_*w*_=120 nm, and *n*=10

The strong dependence of band structure on the spin and valley indices is beneficial to the realization of high spin and valley polarizations. Figure 8 presents the minibands, conductances *G*_*ησ*_, spin polarization *P*_*s*_, and valley polarization *P*_*v*_ as a function of potential *U*. It can be found that the distribution of conductance is completely in agreement with the band structure, that is, the conductance (or conductance gap) corresponds to the miniband (or band gap). The minibands for spin-up and spin-down electrons could be alternative distribution by adjusting *h* properly. Consequently, $G_{K(K^{\prime })\uparrow }$ and $G_{K(K^{\prime })\downarrow }$ present alternative distribution as well, i.e., $G_{K(K^{\prime })\uparrow }$ nearly vanishes for those regions where $G_{K(K^{\prime })\downarrow }$ is in resonance and vice versa. This result directly leads to a remarkable spin polarization, proposing a switching effect of spin polarization (see Fig. 8a). By changing *Δ*_*z*_, the minibands and conductances for electrons near *K* and *K*^′^ valleys could be controlled, leading to a fully valley-polarized current (see Fig. 8b). Compared with spin polarization, the valley polarization is not perfect enough. However, this drawback could be remedied via the disorder structure of the system, as discussed in the following.

**Fig. 9:**
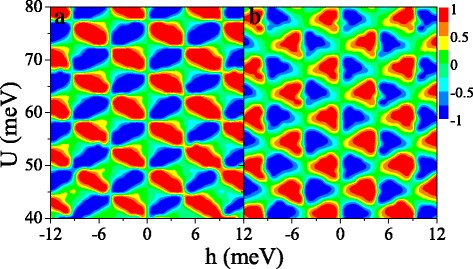
Contour plot of (**a**) spin polarization *P*_*s*_(*U*,*h*) and (**b**) valley polarization *P*_*v*_(*U*,*h*), at *Δ*_*z*_=10.0 meV. The values of other parameters are the same as these in Fig. 8

Figure 9 shows the (a) spin polarization *P*_*s*_ and (b) valley polarization *P*_*v*_ in (*U*,*h*) space. Interestingly, both *P*_*s*_ and *P*_*v*_ present periodical changes in the considered region, which is not observed in the ferromagnetic silicene junction [33]. Both distributions of *P*_*s*_ and *P*_*v*_ are antisymmetric with respect to *h*→−*h*. It is possible to achieve independently a full spin and valley polarization by a proper tuning of the fields *U* and *h*. For example, when *h*=6 meV and *U*=42 meV, *P*_*s*_≈1 and *P*_*v*_≈1, meaning that the current is mainly contributed by *K↑* electrons. When *h*=6 meV and *U*=44 meV, *P*_*s*_≈1 and *P*_*v*_≈−1 while *P*_*s*_≈−1 and *P*_*v*_≈−1 at *h*=6 meV and *U*=46 meV. The results demonstrate that a spin and valley polarization can be switched effectively.

**Fig. 10:**
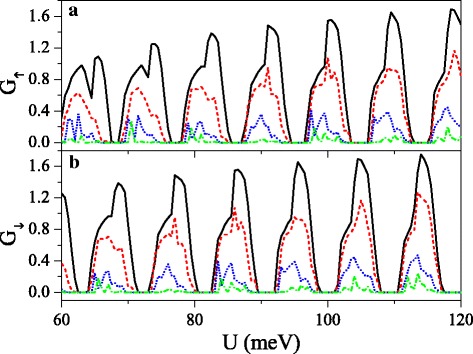
Conductances (**a**) *G*_*↑*_ and (**b**) *G*_*↓*_ versus potential *U*, when the electric field presents disorder, at *n*=50 and *Δ*_*z*0_=20.0 meV. The solid, dashed, dotted, and dash-dotted curves correspond to the disorder strength *δ*=0.0, 0.1, 0.3, and 0.6, respectively. The values of other parameters are the same as these in Fig. 8

**Fig. 11:**
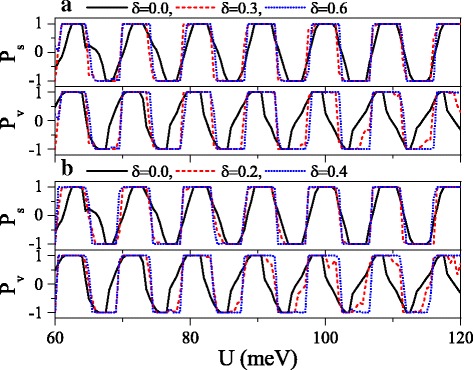
Polarizations *P*_*s*_ and *P*_*v*_ versus potential *U* when **a** the electric field or **b** the exchange field presents disorder. *Δ*_*z*0_=20.0 meV and *h*=7.0 meV in (**a**). *Δ*_*z*_=20.0 meV and *h*_0_=7.0 meV in (**b**). The values of other parameters are the same as these in Fig. 10

In experiment, the structural imperfection of the model is unavoidable due to the limitations of the experimental techniques. Therefore, it is necessary to discuss the effect of the disorder on transmission. When the electric field or exchange field presents disorder, the conductance, spin polarization, and valley polarization are shown in Figs. 10 and 11. We set disorder situations of *Δ*_*z*_ and *h* fluctuate around their mean values, given by 〈*Δ*_*z*_〉=*Δ*_*z*0_ and 〈*h*〉=*h*_0_, respectively. The fluctuations are given by

14 $$\begin{array}{@{}rcl@{}} \Delta_{z} |_{i} = \Delta_{z0} (1 + \delta \zeta_{i}), \quad h |_{i} = h_{0} (1 + \delta \zeta_{i}), \end{array} $$

where {*ζ*_*i*_} is a set of uncorrelated random variables or white noise, − 1<*ζ*_*i*_<1, *δ* is the disorder strength, and *i* is the site index. Note that the disorder only takes place in the *x* direction, and the system is always homogeneous in *y* direction. Thus, *k*_*y*_ still keeps conservation. Figure 10 exhibits the effect of the disorder of the electric field on the conductances (a) *G*_*↑*_ and (b) *G*_*↓*_. With the presence and increase of the disorder strength *δ*, both *G*_*↑*_ and *G*_*↓*_ are suppressed gradually, and each resonant peak splits into many small peaks. One may find that the conductance range is narrowed while the conductance gap range is broadened. Hence, the allowable (or forbidden) ranges of *G*_*↑*_ completely fall into the forbidden (or allowable) ranges of *G*_*↓*_, giving rise to an excellent spin polarization (see Fig. 11). Furthermore, the positions of conductances and conductance gaps are nearly invariable as *δ* changes, suggesting that the miniband and band gap are insensitive to the disorder. Note that the disorder effect of the electric field on *G*_*K*_ and *G*_*K*_^′^ is similar to that observed in Fig. 10. Figure 11 presents the disorder effects of (a) the electric field and (b) the exchange field on polarizations *P*_*s*_ and *P*_*v*_. Obviously, with the increase of *δ*, *P*_*s*_ and *P*_*v*_ increase greatly, and the polarization platform is broadened. Thus, a full spin and valley polarization is realized. Comparison between Fig. 11a and b indicates that the disorder effect of exchange field is more prominent. The results demonstrate that the disorder could enhance the spin and valley polarizations compared with the order case, which is an advantage in realistic application.

## Conclusions

In summary, we demonstrated detailedly that band structure and transport property of silicene under a periodic field strongly depend on the spin and valley degrees of freedom. The numerical results indicate that electrons with different spins and valleys have various characteristics in Dirac point, bang gap, and group velocity. In particular, owing to the electric field and exchange field, the anisotropic velocity is restrained, which displays a collimation behavior for specific spins and valleys. Therefore, the transmission presents strong spin- and valley-dependent feature, consistent with the band structure, resulting in a significant spin and valley polarizations. In addition, the disorder could greatly enhance the spin and valley polarizations. Finally, we hope these results can be conducive to the potential applications of the spin and valley indices.

## Abbreviations

2D: Two-dimensional
FM: Ferromagnetic
SOI: Spin-orbit interaction
